# Identification of Major Risk Sources for Surface Water Pollution by Risk Indexes (RI) in the Multi-Provincial Boundary Region of the Taihu Basin, China

**DOI:** 10.3390/ijerph120810150

**Published:** 2015-08-21

**Authors:** Hong Yao, Weixin Li, Xin Qian

**Affiliations:** 1 State Key Laboratory of Pollution Control and Resource Reuse, School of the Environment, Nanjing University, Nanjing 210023, China; E-Mail: yaohong80@126.com; 2 School of Geography, Nantong University, Nantong 226001, China; 3 Nanjing Institute of Environmental Sciences, Ministry of Environmental Protection of China, Nanjing 210042, China; E-Mail: lwx@nies.org

**Keywords:** risk classification, major risk sources, surface water pollution risk, multi-district boundary region

## Abstract

Environmental safety in multi-district boundary regions has been one of the focuses in China and is mentioned many times in the *Environmental Protection Act* of 2014. Five types were categorized concerning the risk sources for surface water pollution in the multi-provincial boundary region of the Taihu basin: production enterprises, waste disposal sites, chemical storage sites, agricultural non-point sources and waterway transportations. Considering the hazard of risk sources, the purification property of environmental medium and the vulnerability of risk receptors, 52 specific attributes on the risk levels of each type of risk source were screened out. Continuous piecewise linear function model, expert consultation method and fuzzy integral model were used to calculate the integrated risk indexes (RI) to characterize the risk levels of pollution sources. In the studied area, 2716 pollution sources were characterized by RI values. There were 56 high-risk sources screened out as major risk sources, accounting for about 2% of the total. The numbers of sources with high-moderate, moderate, moderate-low and low pollution risk were 376, 1059, 101 and 1124, respectively, accounting for 14%, 38%, 5% and 41% of the total. The procedure proposed could be included in the integrated risk management systems of the multi-district boundary region of the Taihu basin. It could help decision makers to identify major risk sources in the risk prevention and reduction of surface water pollution.

## 1. Introduction

Over the last twenty years, with the rapid development of the economy, multi-district water pollution incidents have risen in China, consequently arousing strong negative social repercussions [1,2,3]. Environmental safety in multi-district regions has been one of the focuses in China and is mentioned many times in an environmental protection law promulgated in 2014 [4]. However, environmental risk analysis for surface water pollution was scarce in these regions [1,5,6].

The Taihu basin is one of the most developed areas in China and encompasses Jiangsu province, Zhejiang province and Shanghai. In the multi-provincial boundary region of the basin, the intensity of economic activities is much greater than other regions and there are a number of national and provincial concentrated industrial zones causing larger pollutant loads [7,8,9]. In addition, this multi-district region is sensitive to environmental pollution and there are several intakes of drinking water suppliers and habitat conservation areas. Environmental safety in the region is not only a matter of security for the population’s normal production and everyday lives, but it is also the foundation for the coordination of upstream and downstream relationships. Presently, eco-compensation, an interdisciplinary topic, has been commonly used to ease multi-district environmental resource competitions [1,10,11,12,13]. The method used in the pilot region of the Taihu basin of China has been well documented [1]. Nonetheless, implementation was far from successful because of the uncertainty of the compensation standard [11]. An enforceable methodology on solving the problem of environmental deterioration in multi-district regions was still lacking [1,5].

The efficient management of pollution sources is most important for the control of surface water pollution [14,15,16,17,18,19]. In the inter-provincial boundary region of the Taihu basin, there are thousands of pollution sources. It is unscientific and unpractical to supervise all sources equally because of financial restrictions in environmental management. Thus to rank and prioritize major sources with high pollution risk is important. Yet there is no quantitative risk characterization method specialized for pollution sources of multi-district regions. Thus the aim of this study is to: (1) develop a quantitative risk characterization method for pollution sources of multi-district regions; (2) rank risk sources and identify major risk sources for surface water pollution in the multi-provincial boundary region of the Taihu basin, China; and (3) explore the contribution of various factors to pollution source risks, so that classification on source risk may be provided for decision makers to set priorities for allocating limited management resources in multi-district regions.

## 2. Materials and Methods

### 2.1. Study Area

**Figure 1:**
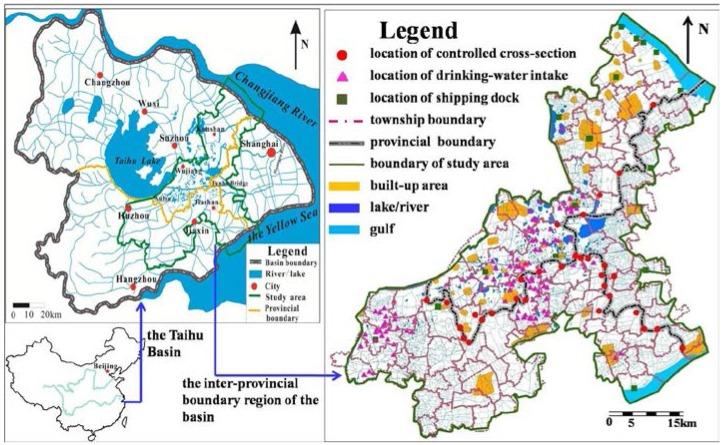
Sketch map of the study area.

The multi-provincial boundary region of the Taihu basin was determined by the region’s contribution to the water quality of national controlled cross-sections on the pollutant loads, which was calculated by water quality model of the basin (Figure 1). The area of the inter-provincial boundary region is about 7712 km^2^ and there are 31 water quality conventional monitoring points controlled by the nation (Figure 1). The region studied has complex river networks. The flow is mainly from west to east and from north to south in Jiangsu province, and from south to north on the border of Zhejiang province and Shanghai. Water quality in the region is in a poor state and none of the 31 points mentioned above could meet the national water quality requirements [20,21].

The region has a lot of economic activities. There are 98 towns and the built-up area is 527 km^2^ (Figure 1). There are 73 centralized waste disposal sites and over 2000 industrial sites. Graded waterways are intensively distributed and 24 large shipping docks are involved in the area (Figure 1). Surface water in the region is sensitive to pollutants and there are 126 drinking-water intake areas in the region. Moreover, the region is densely populated (Figure 1).

### 2.2. Risk Source Types

**Figure 2:**
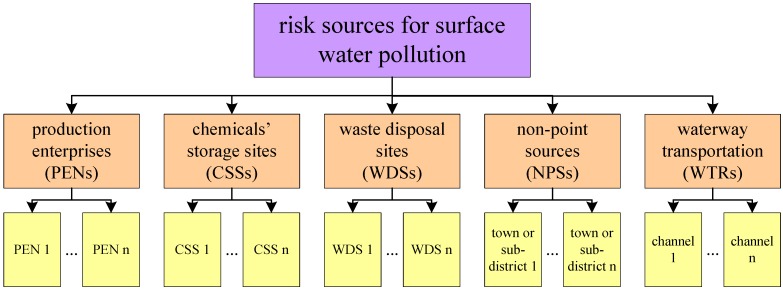
Types of risk sources and units of risk characterization.

Different types of sources for surface water pollution have diverse properties on their risk characteristics and each type should have its own attributes on risk characterization. Based on the source analysis of environmental pollution incidents occurring from 1989 to 2012 in China, five types of pollution sources in the multi-provincial boundary region of the Taihu basin were identified: production enterprises (PENs), chemical storage sites (CSSs), waste disposal sites(WDSs), non-pointsources (NPSs) and waterway transportations (WTSs) (Figure 2).

PENs, CSSs and WDSs were all point sources. The PENs covered all production enterprises with diverse industrial types, sizes, and various environmental management statuses. The CSSs indicated the storage sites of eight kinds of dangerous chemicals defined in the classification of commonly used dangerous chemicals in China (GB13690-1992) [22]. The WDSs included centralized sewage treatment sites and garbage disposal sites. Risk of these three types of sources should be decided on a case-by-case basis (Figure 2). The NPSs indicated the possible negative influence to rivers/lakes imposed by non-point sources’ sewage and the towns or sub-districts in risk assessment units (Figure 2). The channel was the unit of WTRs’ risk characterization (Figure 2).

### 2.3. Overall Procedure of Major Source Identification

Risk of pollution sources is affected by various factors including economic, social, and environmental aspects. Integrated risk index has been reported in many studies and it was commonly obtained by introducing attributes to express the risk levels of pollution sources [6,23,24,25]. The index may truly reflect the overall risk levels of sources if attributes selected are appropriate. For the multi-provincial boundary region of the Taihu basin, which has such specific characteristic as large-scale, complex social and economic conditions, multiple source types and a large number of pollution sources, the way of calculating the risk index to screen out the major risk sources is more feasible and operational [6,9].

**Figure 3:**
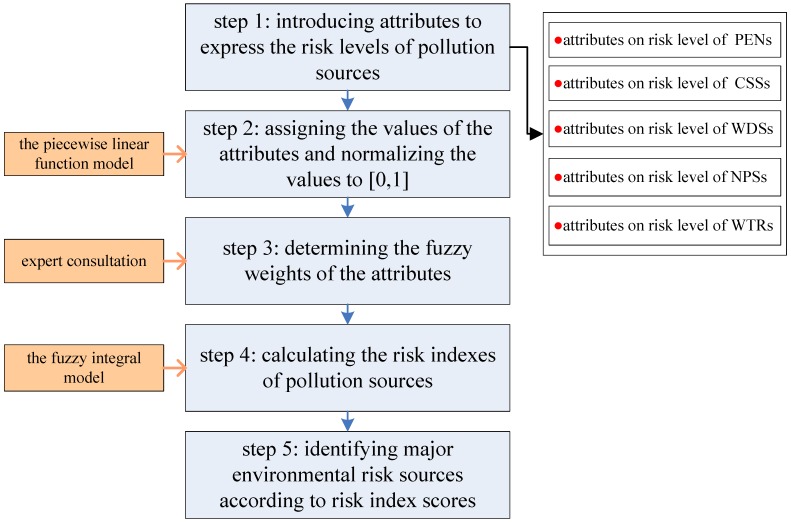
Procedure of major risk source identification by calculating integrated risk indexes.

The procedure includes five steps (Figure 3): (1) introducing attributes to express the risk levels of pollution sources, each type of source having its own attributes; (2) assigning the values of the attributes and normalizing the values to (0, 1) by the piecewise linear function model; (3) determining the fuzzy measure of the attributes by expert consultation; (4) calculating the risk index of pollution sources by the fuzzy integral model; and (5) major environmental risk sources were identified according to risk index scores. Detailed explanations of each of these five steps are provided below.

### 2.4. Attributes on the Risk Levels of Pollution Sources

Sources, environmental mediums and receptors are the three basic elements in environmental risk analysis [9]. They illustrate the whole process of risk exposure: risk factor generation, migration and transformation, ultimately leading to adverse consequences to the environment, humans and the economy [9]. Risk of pollution sources is determined not only by source properties, such as the industrial type, quantity of pollutants discharged, *etc*., but also the hydrodynamic and water quality conditions of the receiving water, and the vulnerability of the corresponding risk receptors [26].

In attribute screening, the adaptability to region-specific contexts, the characteristics of source locations being in the vicinity of the provincial boundaries and data availability are priorities considered. The feasibility of the process of major source identification, including limitations on human resources and financial resources, as well as social and economic development status of the assessed area, is also important in risk management. Thus, when the draft of source attributes came out, we invited several risk managers of the Taihu basin to discuss the attribute items and their grade division criteria. Finally, according to their suggestions, a list of attributes was compiled for each source type (Table 1, Table 2 and Table 3).

Source hazard, environmental medium purification and receptor vulnerability are labeled as X, Y and Z individually. These three factors were respectively characterized by several specific items represented by symbols x_1_, x_2_......, y_1_, y_2_......, and z_1_, z_2_......... Attributes’ items on environmental medium purification and receptor vulnerability of PENs, WDSs and CSSs were the same, so attributes for the three point sources were described in one table (Table 1).

**Table 1:** Attributes of risk levels of PENs, WDSs, CSSs and their grade division criteria.

Attributes(Unit)	Grade Division Criteria			Description and Data Sources
	i	ii	iii	
**PENs-X: source hazard**				
x_1_: the industrial type *	-	-	-	provided by Industry Classification of China(GB/4754-2011) [27]
x_2_: the amount of sewage discharged per year (ten thousand tons)	500	100	20	provided by the latest pollution sources’ census in 2011
x_3_: the amount of chemical oxygen demand discharged per year (ton)	1000	200	20	
x_4_: the amount of ammonia nitrogen is charged per year (ton)	5	2	1	
x_5_: the number of illegal discharges recorded in the past 5years	3	2	0	provided by local environmental protection agencies
**WDSs-X: source hazard**					
x_1_: the type of waste disposed **	hazardous	industrial	others	provided by the latest pollution sources’ census in 2011
x_2_: the amount of sewage discharged per year (ten thousand tons)	1000	200	100	
x_3_: the proportion of working time in the designed time (%)	80	60	30	
x_4_: the number of pollution incidents occurring in the past 5years	3	2	0	provided by local environmental emergency response center
x_5_: the number of illegal discharges recorded in the past 5years	3	2	0	provided by local environmental protection agencies
**CSSs-X: source hazard**					
x_1_: the type of chemicals stored ***	high dangerous	moderate dangerous	mild dangerous	provided by the latest pollution sources’ census in 2011
x_2_: the proportion of working time in the designed time (%)	80	60	30	
x_3_: the number of pollution incidents happened in the past 5years(time)	3	2	0	provided by local environmental protection agencies
x_4_: the location of the CSS assessed	city	suburb	rural	provided by the latest pollution sources’ census in 2011
**Y: environmental medium purification**					
y_1_: the proportion of water quality achieving the targets (%) ^#^	10	50	80	monthly monitoring data [20]
y_2_: the discharge of the river/lake (m^3^/s) ^#^	20	60	100	
**Z: receptor vulnerability**					
z_1_: the distance of the sewage outlet and the controlled cross-section (km)	5	20	30	obtained by the distance calculation tool in ArcGIS
z_2_: the distance of the sewage outlet and the drinking water intake (km) ^##^	5	20	30	
z_3_:the number of rare aquatic species in the surface water ^##^	3	2	0	provided by statistical yearbooks of the counties
z_4_: population density (/km^2^) ^##^	1500	1100	500	

* “x_1_: the industrial type”: the most hazardous industry (first level) denotes chemical, electroplating, leather, dyeing and paper production industries; the second hazard level denotes textile, food processing industries; and the third level is machinery manufacturing industry and others; ** “hazardous waste” denotes the ones listed in National Hazardous Waste Items of China(GB 12268-90); *** “high dangerous chemicals” denote substances listed as class six to eight in National Dangerous Chemicals Items of China(GB 12268-90); moderate ones denote the fifth class and mild ones denote class one to class four; ^#^ “the proportion of water quality achieving the targets” denotes the statistical result of the monthly water quality monitoring data *in stiu* in the past one year; “the discharge of the river/lake” denotes the average of the monthly hydrologic data in the past ten year; ^##^ “the drinking water intake” denotes the nearest drinking water intake downstream the source’s sewage outlet; “the number of rare aquatic species” denotes aquatic wildlife under national or local protection; “population density” denotes population density of the contribution region to the water quality of the corresponding controlled cross-section.

**Table 2:** Attributes of risk levels of NPSs and their grade division criteria.

Attributes(Unit)	Grade Division Criteria			Description and Data Sources	
	i	ii	ii		
**X: source hazard**					
x_1_: estimation of COD into the water from the NPS (kg/day)	8000	4000	1000	the pollution load of the corresponding non-point sources to the rivers/lakes, which was estimated using pollutants’ export coefficients [28,29]	
x_2_: estimation of total nitrogen into the water from the NPS (kg/day)	1000	600	200		
x_3_: estimation of total phosphorus into the water from the NPS (kg/day)	250	150	50		
**Y: environmental medium purification**					
the same as Y in Table 1					
**Z: receptor vulnerability**					
z_1_:the distance of the NPS’s center and the controlled cross-section (km)	5	20	30	obtained by the distance calculation tool in ArcGIS	
z_2_: the percentage of the drinking water source protection area in the whole area of the receiving water (%)	2	1.2	0	obtained by the area calculation tool in ArcGIS	
z_3_: the number of rare aquatic species in the water	the same as z_3_ and z_4_ in Table 1				
z_4_: population density (/km^2^)					

**Table 3:** Attributes of risk levels of WTRs and their grade division criteria.

Attributes(unit)	Grade Division Criteria			Description and Data Sources	
	i	ii	iii		
**X: source hazard**					
x_1_: the shipping number of the channel (/day)	2000	1200	200	provided by the transportation Yearbook of the Taihu basin [30,31]	
x_2_: the shipping number with hazardous chemicals (/day)	100	60	20		
x_3_: the number of refueling points in the channel	10	6	2	Ibid. refueling points denote the ones with capacity y>300 tons; loading docks with capacity > 300 tons	
x_4_: the number of loading docks in the channel	10	6	2		
**Y: environmental medium purification**					
the same as Y in Table 1					
**Z: receptor vulnerability**					
z_1_: the nearest distance of the controlled cross-section and refueling points (km)	5	20	30	obtained by the distance calculation tool in ArcGIS	
z_2_: the nearest distance of the controlled cross-section and loading docks (km)	5	20	30		
z_3_: the percentage of the drinking water source protection area in the whole channel (%)	2	1.2	0	obtained by the area calculation tool in ArcGIS	
z_4_: the number of rare aquatic species in the water	the same as z_3_ and z_4_ in Table 1				
z_5_: population density (/km^2^)					

The number of identified attributes is not suitable for too much because attributes have to be aggregated on the basis of experts’ insights in the later process, which are not easily elicited when the number of attributes is too large. In source hazard characterization, the amounts of sewage and pollutants discharged were all considered: “the number of illegal discharging recorded in the past 5 years” and “the number of environmental pollution incidents happened in the past 5 years” were used to indicate risk management status of the sources. In environmental medium analysis, “the discharge of the pollutants’ receiving river/lake” and “the proportion of water quality achieving the targets” reflected their capacities of receiving pollutants. In receptor vulnerability analysis, water quality of the national controlled cross-sections and drinking water intakes, the numbers of rare aquatic species and residents were all involved.

### 2.5. Attribute Values Assignment and Their Normalization

Each item in Table 1, Table 2 and Table 3 was divided into three grades—i, ii and iii—which respectively represented high, moderate and low pollution risk. All values assignment complied with the conservative principles, and in the condition of various values occurring in one attribute, the value representing higher risk class was used.

After the attribute values assignment step, normalization procedure was performed to convert all attributes into the same domain and the codomain proposed was a (0, 1) interval. All the normalization scores and classification of indicators were referred to the references or defined by experts’ judgment [32]. The discrete values, such as “the industrial type” (x_1_ of the PENs-X), “the location of the source” (x_4_ of the CSSs-X), were directly normalized to a score in (0, 1) according to their risk grades and the normalized scores of grades i to iii were 1, 0.6 and 0.2, respectively. The continuous piecewise linear model, which used diverse liner functions to calculate normalized scores in different intervals, was proposed to be the scoring function in normalizing the attributes characterized by continuous values [32]. Points of segmentation were presented in Table 1, Table 2 and Table 3 as the grade division criteria of attributes.

For positive attributes such as “population density”, the higher the values of these indicators, the higher the risk. According to the piecewise linear model, the normalized scores of these attributes were obtained by the following formula and A in the expression represented the attribute value assigned:

If A ≥ grade i, normalized score = 1;
If grade ii ≤ A < grade i, normalized score = 1 − 0.4 × (grade i − A)/(grade i − grade ii);
If grade iii < A < grade ii, normalized score = 0.6 − 0.4 × (grade i − A)/(grade ii − grade iii);
If A ≤ grade iii, normalized score = 0.2.

For negative attributes, such as “the distance of the sewage outlet and the controlled cross-section,” the higher the values of these indicators, the lower the risk. The normalized scores of these attributes were obtained by the following formula and B in the expression represented the attribute value assigned:

If B ≤ grade i, normalized scores = 1;
If grade i < B ≤ grade ii, normalized scores = 1 − 0.4× (B-grade i)/(grade ii − grade i);
If grade ii < B < grade iii, normalized scores=0.6 − 0.4 × (B-grade ii)/(grade iii − grade ii);
If B ≥ grade iii, normalized scores = 0.2.

After this process, all normalization values of attributes were in the same closed interval (0, 1).

### 2.6. Fuzzy Measure Scores of Attribute Coalitions

Risk estimation is performed by aggregating the sources’ normalized attribute values. This subjective evaluation process commonly shows nonlinear characteristics and implications expressed by indicators overlapping. Discrete choquet fuzzy integral, one nonlinear function, is suitable for measuring the importance of indicators; the method has been successfully used in risk analysis [32,33].

X = (x_1_, x_2_……x_n_), Y= (y_1_, y_2_……y_m_) and Z = (z_1_, z_2_……z_p_) have been defined as risk attribute sets in Section 2.4 (Table 1, Table 2 and Table 3). T = (X, Y, Z), the coalition of source hazard, environmental medium purification and receptor vulnerability, is the risk level of pollution sources.

In analyzing source hazard, different coalitions of attributes, that is the power set P(X) of the attributes set X, have different contributions on the hazard of sources. Fifteen experts involved in water environmental management of the Taihu basin were invited to participate in the questionnaires on evaluating the scores for each coalition of attributes [34]. Scores were required to be in the (0, 1) interval; 1 denotes the highest class potentially generating pollution hazard and 0 expresses the lowest hazard. The average values derived from the experts were the fuzzy measure scores for P(X).

Similarly, the fuzzy measure scores for the power set P(Y) of the attributes set Y, the power set P (Z) of the attributes set Z and the power set P(T) of the attributes set T could also be obtained. For simplification, here we only presented fuzzy measure scores of various indicators coalitions for the NPSs (Table 4).

**Table 4:** Fuzzy measure scores of various indicators coalitions for the NPSs.

X				Y			Z					T			
x_1_	x_2_	x_3_	Score	y_1_	y_2_	Score	z_1_	z_2_	z_3_	z_4_	Score	X	Y	Z	Score
0	0	0	0	0	0	0	0	0	0	0	0	0	0	0	0
1	0	0	0.41	1	0	0.53	1	0	0	0	0.60	1	0	0	0.41
0	1	0	0.38	0	1	0.50	0	1	0	0	0.34	0	1	0	0.40
0	0	1	0.44	1	1	1	0	0	1	0	0.32	0	0	1	0.45
1	1	0	0.72				0	0	0	1	0.26	1	1	0	0.70
1	0	1	0.67				1	1	0	0	0.75	1	0	1	0.83
0	1	1	0.71				1	0	1	0	0.72	0	1	1	0.72
1	1	1	1				1	0	0	1	0.70	1	1	1	1
							0	1	1	0	0.58				
							0	1	0	1	0.60				
							0	0	1	1	0.56				
							1	1	1	0	0.87				
							1	1	0	1	0.85				
							1	0	1	1	0.82				
							0	1	1	1	0.79				
							1	1	1	1	1				

### 2.7. Risk Index

Discrete choquet fuzzy integral algorithm was used to calculate the risk indexes of the pollution sources [32,33]. “h1” is defined as the mapping functions of the set X to (0, 1) interval and this mapping indicates the process of the attribute values normalization. “h2” represents all the different normalization functions for the different criteria in Y and “h3” denotes those in Z. The elements in X, Y and Z were then resorted to a new sequence denoted as ${\text{X}}^{\prime }$ = {${\text{x}}_{1}^{\prime }$, ${\text{x}}_{2}^{\prime }$…${\text{x}}_{\text{n}}^{\prime }$}, ${\text{Y}}^{\prime }$ = {${\text{y}}_{1}^{\prime }$, ${\text{y}}_{2}^{\prime }$…${\text{y}}_{\text{m}}^{\prime }$}, ${\text{Z}}^{\prime }$ = {${\text{z}}_{1}^{\prime }$, ${\text{z}}_{2}^{\prime }$…${\text{z}}_{\text{p}}^{\prime }$}, to make h1(${\text{x}}_{1}^{\prime }$) ≥ h1(${\text{x}}_{2}^{\prime }$) ≥…≥ h1 (${\text{x}}_{\text{n}}^{\prime }$), h2(${\text{y}}_{1}^{\prime }$) ≥ h2(${\text{y}}_{2}^{\prime }$) ≥…≥ h2 (${\text{y}}_{\text{m}}^{\prime }$), h3(${\text{z}}_{1}^{\prime }$) ≥ h3(${\text{z}}_{2}^{\prime }$) ≥…≥h3(${\text{z}}_{\text{p}}^{\prime }$). “g1”, “g2” and “g3” are defined as the mapping functions of various indicators coalitions of X, Y and Z to their fuzzy measure scores.

Thus discrete choquet fuzzy integral of fuzzy measure g and indicator normalization evaluation score h were used to express source hazard index (HI), environmental medium purification index (EI) and receptor vulnerability index (VI). The formula of calculating HI is listed below and is the same for EI and VI:

$$\begin{array}{c} \text{HI}=\int^{\text{​}}\text{h}1\text{dg}1=\text{h}1\left({\text{x}}_{\text{n}}^{\prime }\right)\text{g}1\left({\text{X}}_{\text{n}}^{\prime }\right)+\left(\text{h}1\left({\text{x}}_{\text{n}-1}^{\prime }\right)-\text{h}1\left({\text{x}}_{\text{n}}^{\prime }\right)\right)\text{g}1\left({\text{X}}_{\text{n}-1}^{\prime }\right)+\ldots \left(\text{h}1\left({\text{x}}_{i-1}^{\prime }\right)-\text{h}1\left({\text{x}}_{i}^{\prime }\right)\right)\text{g}1\left({\text{X}}_{i-1}^{\prime }\right)+\ldots +\left(\text{h}1\left({\text{x}}_{1}^{\prime }\right)-\text{h}1\left({\text{x}}_{2}^{\prime }\right)\right)\text{g}1\left({\text{X}}_{1}^{\prime }\right)\\ \text{where: }{\text{X}}_{i}^{\prime }=\left\{{\text{x}}_{1}^{\prime },{\text{x}}_{2}^{\prime },\ldots ,{\text{x}}_{i}^{\prime }\right\} \end{array}$$

With the proposed method, HI, EI, VI values could be obtained. The three variables were all in the interval (0, 1). The higher the HI value was, the more hazardous the pollution source was. The higher the EI value was, the worse the purification capacity of the surface water was. The higher the VI value was, the more vulnerable the receptor was.

“h4” is defined as the mapping function of the set T = (X, Y, Z) to (0, 1) interval, and this mapping indicates the values of HI, EI and VI obtained above. The elements in T are then resorted to a new sequence denoted as ${\text{T}}^{\prime }$ = (${\text{t}}_{1}^{\prime }$, ${\text{t}}_{2}^{\prime }$, ${\text{t}}_{3}^{\prime }$) to make h4 (${\text{t}}_{1}^{\prime }$) ≥ h4 (${\text{t}}_{2}^{\prime }$) ≥ h4 (${\text{t}}_{3}^{\prime }$). “g4” is defined as the mapping function of indicator coalitions of T to their fuzzy measure scores. Thus risk indexes of pollution sources were obtained by the expression:

$$\text{RI}=\begin{array}{c} \int^{\text{​}}\text{h}4\text{dg}4=\\ \;\text{h}4\left({\text{t}}_{3}^{\prime }\right)\text{g}4\left({\text{T}}_{3}^{\prime }\right)+\left(\text{h}4\left({\text{t}}_{2}^{\prime }\right)-\text{h}4\left({\text{t}}_{3}^{\prime }\right)\right)\text{g}4\left({\text{T}}_{2}^{\prime }\right)+\left(\text{h}4\left({\text{t}}_{1}^{\prime }\right)-\text{h}4\left({\text{t}}_{2}^{\prime }\right)\right)\text{g}4\left({\text{T}}_{1}^{\prime }\right)\\ \text{Where: }{\text{ T}}_{\text{i}}^{\prime }=\left\{{\text{t}}_{1}^{\prime },\ldots ,{\text{t}}_{\text{i}}^{\prime }\right\} \end{array}$$

RI, the comprehensive representation of pollution source hazard, surface water purification and receptor vulnerability, was used to measure the risk levels of the pollution sources. The higher the RI value, the higher the potential risk of the source.

### 2.8. Risk Classification and Major Risk Source Identification

**Figure 4:**
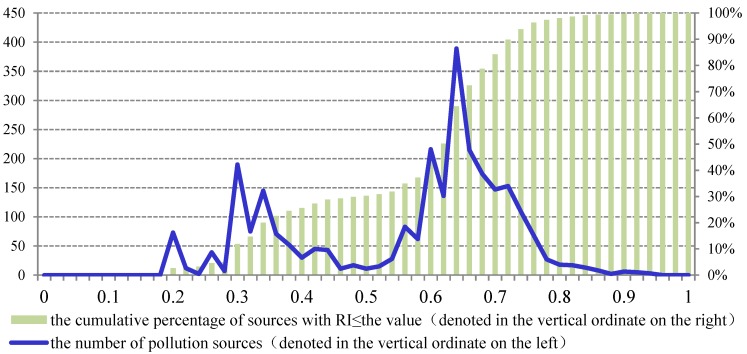
The distribution of RI values of all pollution sources in the study area.

In the multi-provincial boundary region of the Taihu basin, 2716 pollution sources were characterized by risk indexes. The majorities of the sources’ RI values were in 0.6–0.8 and 0.3–0.4 (Figure 4). In the figure, the horizontal ordinate denoted RI values of risk sources, which were in 0–1. Considering the environmental risk management aims and resource limitations of the region, sources were divided into five categories: high risk, high-moderate risk, moderate risk, moderate-low risk and low risk. The classification criteria were identified by the cumulative proportion of each class (Table 5).

Thus major risk sources could be identified according to the result of risk classification, and sources with high pollution risk were regarded as major risk sources in environmental risk management of the study area.

**Table 5:** Risk classification criteria and the proportion of each class of sources.

Risk Classification	Classification Criteria	Number of Sources (Proportion)	PENs	CSSs	WDSs	NPSs	WTSs
high risk	RI > 0.8	56 (2%)	21	0	2	27	6
high-moderate risk	0.7 < RI ≤ 0.8	376 (14%)	250	82	28	12	4
moderate risk	0.6 < RI ≤ 0.7	1059 (38%)	874	150	23	10	2
moderate-low risk	0.5 < RI ≤ 0.6	101 (5%)	36	15	9	41	0
low risk	RI ≤ 0.5	1124 (41%)	1025	80	11	8	0
Total		2716 (100%)	2206	327	73	98	12

## 3. Results and Discussion

Multiple attributes of the pollution sources were investigated and information on hydrological parameters and water qualities in the rivers of the provincial boundary region of the Taihu basin were also monitored *in situ*. All data, including those of pollution sources, environmental medium, risk receptors and the fuzzy measure scores of various attribute coalitions were all introduced into the risk source information database. The corresponding relationships of pollution sources, environmental medium and risk receptors were connected and could be queried in the database. Data sources of all attributes were listed in Table 1, Table 2 and Table 3. Then the risk levels of all pollution sources were characterized by the procedure proposed above.

### 3.1. Production Enterprises (PENs)

**Figure 5:**
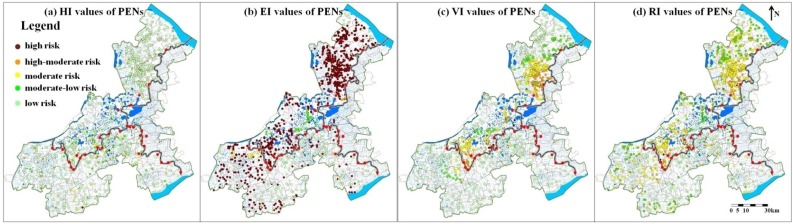
Spatial distribution of HI, EI, VI and RI values of the PENs.

A total of 2206 industrial sources discharging disposed waste water into surface water directly were assessed. There were 1376 enterprises located in Jiangsu province and 830 were in Zhejiang province. Kunshan and Wujiang of Jiangsu province, two cities with well-developed industries, were enterprise-concentrated areas (Figure 5).

The maximum value of risk indexes of the PENs was 0.86 and the minimum value was 0.2. There were 21 high-risk enterprises screened out as major risk sources, accounting for about 1% of the total PENs. The numbers of the enterprises with high-moderate, moderate, moderate-low and low pollution risk were 250, 874, 36 and 1025, respectively, accounting for 11%, 40%, 2% and 46% of the total PENs. In total, more than half (52%) of the PENs in the multi-provincial boundary area were in the more serious pollution risk category than in the moderate level, and these enterprises were mainly distributed in the vicinity of the provincial boundaries (Figure 5d). The majorities of the remaining ones were in low pollution risk.

HI, EI and VI values were also presented to illustrate the reasons for the pollution risk of sources (Figure 5a–c). The grading criteria of the three indexes were the same as that of RI. In general, the hazard of the PENs were mainly in low level (HI < 0.5) and environmental medium purification were rather poor (EI > 0.8). Spatial distribution of receptor vulnerability was similar with that of the PENs’ risk levels and, in the vicinity of the provincial boundaries, receptors were more vulnerable. From the distribution of HI, EI, VI and RI values, we can conclude that the pollution risk of these enterprises was attributed to poor purification of surface water and vulnerable risk receptors. The former factor originated mainly from the low flow and poor water quality of surface water [9]. The vulnerabilities of receptors were due to their locations near the multi-provincial boundaries and densely distributed drinking water intakes.

The 21 major risk PENs, which were located in Kunshan and Wujiang of Jiangsu province, all belonged to such heavy pollution industries as chemical and paper production, leather, dyeing, *etc.* These major risk sources were all in the vicinity of the provincial boundaries and the pollutants’ receiving water had at least one drinking water intake. Furthermore, these major risk sources were all the biggest producers of pollutants. The sewage emissions of the 21 PENs accounted for about 12% of the emissions of all the enterprises in the study area. The amounts of chemical oxygen demand and the ammonia nitrogen emissions were about 14% and 15% of all the PENs. Thus the 21 enterprises identified as major risk sources truly need to be the focus in the prevention and control of surface water pollution risk in the region.

### 3.2. Chemical Storage Sites (CSSs)

**Figure 6:**
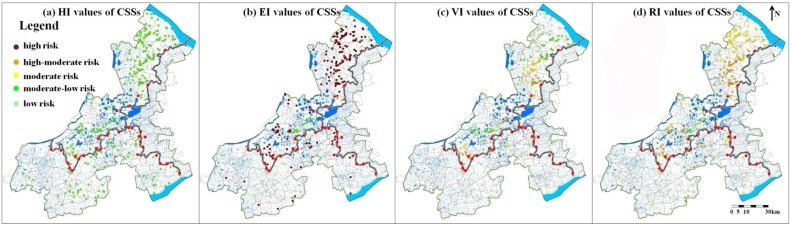
Spatial distribution of HI, EI, VI and RI values of the CSSs.

Chemical storage information of 327 sites was obtained. These sites were mainly in Jiangsu province (Figure 6). The maximum value of risk indexes of these CSSs was 0.78 and the minimum value was 0.33. Of the five risk classes, the CSSs with moderate pollution risk had the biggest proportion. The number of this kind of CSSs was 150, accounting for 46% of all the CSSs. The numbers of high-moderate risk and low risk CSSs were respectively 82 and 80 with the portions of 25% and 24%. The remaining 15 sites had moderate-low pollution risk, sharing 5% of the CSSs. Though there is no high-risk site (major risk source) with RI > 0.8, the CSSs with the pollution risk more serious than the moderate level (RI > 0.6) accounted for the majority of the total and shared the proportion of 71%, which is bigger than that of the PENs.

Similar to the PENs, the CSSs with higher pollution risk were mainly located in the vicinity of the provincial boundaries (Figure 6d). The distribution of HI, EI, VI values were also similar to that of the PENs (Figure 6a–c). The hazards of the CSSs were mainly in moderate-low levels, environmental medium purification were rather poor, and spatial distribution of receptor vulnerability was similar with that of CSS risk. Similar conclusions with PENs could be also obtained that the pollution risk of chemical storage sites was mainly attributed to the four factors: the low flow and the poor water quality of the receiving water, source locations close to the multi-district boundaries and the dense distribution of drinking water intakes.

### 3.3. Waste Disposal Sites (WDSs)

**Figure 7:**
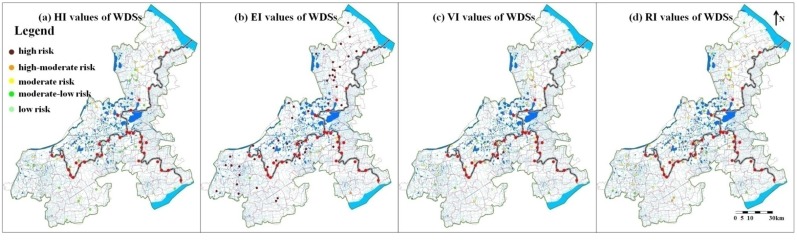
Spatial distribution of HI, EI, VI and RI values of the WDSs.

The information for 73 waste disposal sites was obtained. Of these, 44 were in Jiangsu province and 29 were in Zhejiang province. They were all evenly distributed in the region (Figure 7). Waste of the 73 sites were all waste water, including untreated urban sewage and industrial effluent.

The maximum value of risk indexes of these WDSs was 0.81 and the minimum value was 0.2. RI values of the WDSs were evenly distributed. Two WDSs had high pollution risk and major risk WDSs hold about 3% of the total WDSs. The numbers of the WDSs with high-moderate, moderate, moderate-low and low pollution risk were 28, 23, 9 and 11, respectively, thereby accounting for 38%, 31%, 12% and 16% of the WDSs. In total, about 72% of the WDSs had a pollution risk more serious than the moderate level and this percentage was close to that of the CSSs.

The distribution of HI, EI, VI and RI values were also similar to those of the PENs and the CSSs (Figure 7). The WDSs were mainly low hazards (HI < 0.5), and environmental medium purification was rather poor (EI > 0.8). Similar conclusions could also be made for pollution risk of waste disposal sites being attributed to the four factors mentioned in Section 3.1 and Section 3.2.

### 3.4. Non-Point Sources (NPSs)

**Figure 8:**
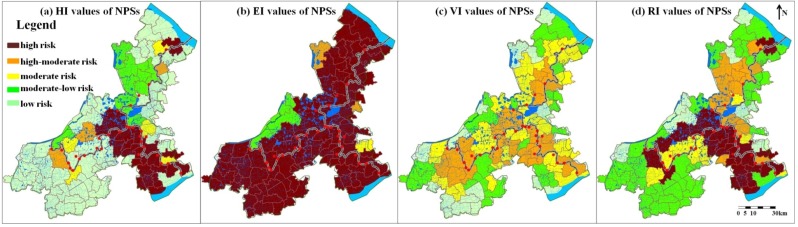
Spatial distribution of HI, EI, VI and RI values of the NPSs.

Information of 247 assessment units of non-point sources was introduced into the risk source database. Because pollutants discharged by some towns and sub-districts might flow into many rivers or lakes and pose problems for diverse surface water, these pollution sources corresponded to multiple environmental medium and risk receptors and furthermore had multiple RI values. Based on the conservative principle in risk assessment, the highest RI value, representing the most serious risk status, was identified as the risk level of the non-point source. Finally, RI values of 98 towns and sub-districts were confirmed (Figure 8).

The maximum RI value of the NPSs was 0.93 and the minimum value was 0.37. Of the 98 non-point sources, 27 NPSs had high pollution risk and the major risk NPSs hold about 27% of the total NPSs. This proportion was much higher than those of point sources. This was consistent with some conclusions reported [6,35,36]. The numbers of the NPSs with high-moderate, moderate, moderate-low and low pollution risk were 12, 10, 41 and 8, respectively accounting for 12%, 10%, 42% and 8% of all the NPSs.

I total, half of the NPSs in the area were had pollution risk more serious than the moderate level, which distributed along the provincial boundaries (Figure 8d). Of the three provincial boundaries, the region along the boundary of Zhejiang province and Shanghai had the highest risk of non-point source pollution and was concentrated with high-risk NPSs, followed by that of Zhejiang province and Jiangsu province. The area along the boundary of Jiangsu province and Shanghai was the lowest (Figure 8d). Moderate-low and low risk NPSs were all towns or sub-districts far away from the provincial borders.

In general, source hazard, environmental medium purification and receptor vulnerability of the NPSs all had significant influence on risk levels of the NPSs (Figure 8a–c). Environmental medium purification was poor (Figure 8b), the hazard of NPSs and receptor vulnerability both had obvious spatial heterogeneity and shared spatial distribution characteristics with the RI values (Figure 8a,c). Thus the high pollution risk of non-point sources could originate from the five factors: the high pollution load, the low flow of the water, the poor water quality, sources being at the sensitive locations and the densely distributed drinking water intakes.

### 3.5. Waterway Transportation (WTSs)

Information on 12 high-grade waterways was collected, and the length of the waterways added up to 595 km.

The maximum RI value was 0.89 and the minimum was 0.67. Of the 12 WTSs, six channels were identified to be major risk sources, and high risk WTSs shared about 50% of the total. This proportion was the highest of all source categories. The numbers of the WTSs with high-moderate and moderate pollution risk were 4 and 2, respectively, accounting for 33% and 7% of all the WTSs. Thus all waterways assessed had a pollution risk more serious than the moderate level.

**Figure 9:**
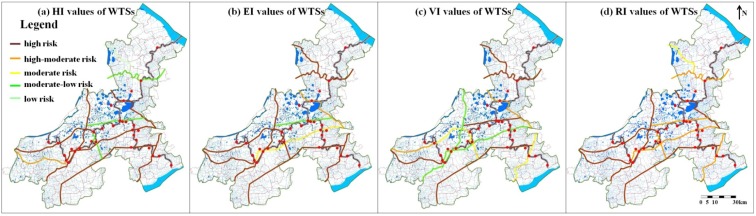
Spatial distribution of HI, EI, VI and RI values of the WTSs.

The four indicators, HI, EI, VI and RI values, all presented spatial heterogeneity (Figure 9). The hazards of the WTSs and receptor vulnerabilities were all high, and environmental medium purification was poor. The three factors all had significant influence on risk levels of the WTSs. Thus the high pollution risk of waterways could originate from multiple factors: the high shipping number, the densely distributed refueling points and loading docks, the low flow and the poor water quality of the water, sources being at the pollution-sensitive locations and the densely distributed drinking water intakes.

Lower grade waterways (below grade six) were also important in the prevention and control of waterway pollution risk. Many waterways were below grade six in the region and for those in places such as Jiashan County, the number was as high as 53. Presently, information on this kind of channel is difficult to obtain. In future work, if data on these sources becomes available, risk assessment and major risk source identification should be also carried out.

## 4. Conclusions

Risk sources of surface water pollution in the multi-provincial boundary region of the Taihu basin were categorized into five types. Considering the hazards of risk sources, the purification properties of surface water and the vulnerabilities of risk receptors, one procedure for risk source classification and major risk source identification was proposed and 52 attributes on the risk levels of pollution sources were screened out. After the fuzzy measure scores of attribute coalitions were assigned, the fuzzy integral model was used to calculate risk indexes (RI), which were used to determine the risk levels of the pollution sources. A total of 2716 pollution sources, including 2206 production enterprises, 327 chemical storage sites, 73 waste disposal sites, 98 non-point sources and 12 waterway transportations, were characterized by RI values. There were 56 high-risk sources screened out as major risk sources, accounting for about 2% of the total. The numbers of sources with high-moderate, moderate, moderate-low and low pollution risk were 376, 1059, 101 and 1124, respectively, thereby accounting for 14%, 38%, 5% and 41% of all sources.

The results of this assessment will be important to help decision makers interpret source risk in specific regions. In addition, the procedure proposed could rapidly reclassify all sources if source information changes. Thus the automation of the whole process, including classifying risk sources, major risk source identification and high-risk source interpretation in the region, were realized.
